# Elevated plasma syndecan-1 as glycocalyx injury marker predicts unfavorable outcomes after rt-PA intravenous thrombolysis in acute ischemic stroke

**DOI:** 10.3389/fphar.2022.949290

**Published:** 2022-07-15

**Authors:** Fangfang Zhao, Rongliang Wang, Yuyou Huang, Lingzhi Li, Liyuan Zhong, Yue Hu, Ziping Han, Junfen Fan, Ping Liu, Yangmin Zheng, Yumin Luo

**Affiliations:** ^1^ Institute of Cerebrovascular Disease Research and Department of Neurology, Xuanwu Hospital of Capital Medical University, Beijing, China; ^2^ Beijing Geriatric Medical Research Center and Beijing Key Laboratory of Translational Medicine for Cerebrovascular Diseases, Beijing, China; ^3^ Beijing Institute for Brain Disorders, Capital Medical University, Beijing, China

**Keywords:** syndecan-1, acute ischemic stroke, rt-PA, prognosis, glycocalyx

## Abstract

**Purpose:** We aimed to examine the prognostic value of syndecan-1 as a marker of glycocalyx injury in patients with acute ischemic stroke (AIS) receiving rt-PA intravenous thrombolysis.

**Methods:** The study included 108 patients with AIS treated with rt-PA intravenous thrombolysis and 47 healthy controls. Patients were divided into unfavorable and favorable prognosis groups based on modified Rankin Scale scores. Univariate and multivariate logistic regression analyses were used to determine risk factors affecting prognosis. Risk prediction models presented as nomograms. The predictive accuracy and clinical value of the new model were also evaluated.

**Results:** Plasma levels of syndecan-1 were significantly higher in patients with AIS than in controls (*p* < 0.05). Univariate analysis indicated that higher levels of syndecan-1 were more frequent in patients with poor prognosis than in those with good prognosis (t = −4.273, *p* < 0.001). Syndecan-1 alone and in combination with other factors predicted patient outcomes. After adjusting for confounding factors, syndecan-1 levels remained associated with poor prognosis [odds ratio, 1.024; 95% confidence interval (CI), 1.010–1.038]. The risk model exhibited a good fit, with an area under the receiver operating characteristic curve of 0.935 (95% CI, 0.888–0.981). The categorical net reclassification index (NRI) and continuous NRI values were >0. The integrated discrimination improvement value was 0.111 (95% CI, 0.049–0.174, *p* < 0.001). Decision curve analysis indicated that the model incorporating syndecan-1 levels was more clinically valuable than the conventional model.

**Conclusion:** Plasma syndecan-1 levels represent a potential marker of prognosis of AIS following intravenous thrombolysis. Adding syndecan-1 to the conventional model may improve risk stratification.

## Introduction

Ischemic stroke is the leading cause of disability and the third leading cause of death worldwide, following heart disease and cancer, accounting for approximately 5.5 million deaths globally, two-thirds of which occur in developing countries. In addition, many survivors with ischemic stroke live with disabilities. Treatment for acute ischemic stroke (AIS) involves restoring perfusion to the affected cerebral area (i.e., reperfusion therapy) as soon as possible. Recombinant tissue plasminogen activator (rt-PA), an ischemic stroke treatment approved by the Food and Drug Administration, has been shown to increase the number of patients with good prognosis by 11%–13% when administered within 3 h of AIS onset. The same study conducted by the National Institute of Neurological Disorders and Stroke reported that tPA treatment reduced the frequency of disability and death at 3 months post-AIS (National Institute of Neurological Disorders and Stroke rt-PA Stroke Study Group, 1995). Subsequently, the time window for administering intravenous thrombolytic therapy with rt-PA was extended to 4.5 h, which remained associated with significant improvements in clinical outcomes.

Despite its demonstrated efficacy, rt-PA has adverse effects, including brain edema and intracranial hemorrhage (Tsuji et al., 2005). A growing body of evidence suggests that tPA mediates increases in blood–brain barrier permeability induced by cerebral ischemia (Yepes et al., 2003; Zhu et al., 2019). Animal experiments have also demonstrated that tPA treatment can induce Evans dye extravasation, increasing the expression and activity of matrix metallopeptidase 9 (MMP-9) (Zhu et al., 2019), which has been associated with late neurotoxicity (Yepes et al., 2003).

Recent studies have identified endothelial glycocalyx as an integral part of the expanded neurovascular unit given its important role in maintaining neuronal homeostasis (Stanimirovic and Friedman, 2012). Endothelial glycocalyx is composed of proteoglycan and GAG chains (van Vliet et al., 2014), forming a barrier between the blood and vessels. Notably, injury to endothelial glycocalyx appears to be the first step in blood–brain barrier dysfunction (Wang et al., 2016). Syndecans are members of the transmembrane heparan sulfate glycoprotein family that are mainly expressed on the surface of cells, including vascular endothelial cells (Chronopoulos et al., 2020). In addition, their complete extracellular domains can be shed into the extracellular environment (Faria-Ramos et al., 2021). Changes in the expression or distribution of syndecan-1 may affect the integrity of the endothelial glycocalyx and endothelial barrier function (Kim et al., 2020). Increased levels of syndecan-1 in the blood may also represent a marker of glycocalyx degradation (Yao et al., 2019), and decreased thickness of the endothelial glycocalyx has been reported in syndecan-1^−/−^ mice (Lu et al., 2020). In this study, we aimed to examine the prognostic value of syndecan-1 levels as a marker of glycocalyx injury after thrombolysis in patients with AIS.

## Methods

**FIGURE 1:**
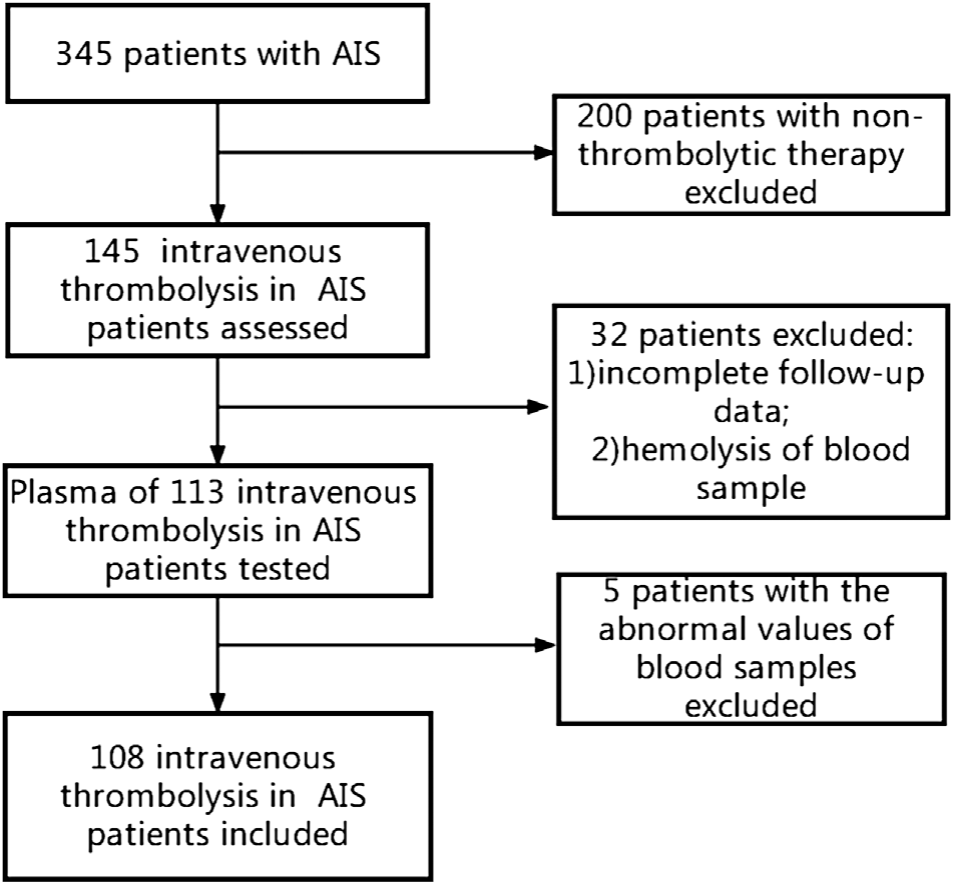
Study flow chart.

This cohort study included 345 patients with acute cerebral infarction who had presented to Xuanwu Hospital of Capital Medical University within 24 h of onset between September 2018 and May 2019. A total of 145 patients received intravenous thrombolysis with rt-PA. Thirty-two patients were excluded due to incomplete medical records, incomplete follow-up data, or blood sample hemolysis. Data were analyzed for 113 patients who had received thrombolytic therapy; five patients were excluded due to abnormal plasma test results. Thus, the final analysis included 108 patients (Figure 1). A total of 47 age- and sex-matched healthy participants were included in the control group. This study was approved by the Ethics Committee of the Xuanwu Hospital of Capital Medical University.

Patients were eligible for this study if they met the following criteria: diagnosis of AIS, as defined by the relevant Chinese guidelines (Zhong et al., 2019); absence of low-density changes on brain computed tomography scans; willingness to participate and provide informed consent; clear neurological deficits; onset time <4.5 h; no previous cerebral infarction; and complete case and follow-up data. Exclusion criteria were as follows: history of cerebral infarction; diagnosis of hemorrhagic disease; diagnosis of malignant tumors, blood disease, or severe infection; pregnancy; diagnosis of epilepsy, mental illness, coagulation disorders, or liver/kidney dysfunction.

### Collection of clinical data and blood samples

Patients with AIS were treated with rt-PA at a dose of 0.9 mg/kg. An intravenous injection containing 10% of the total amount of the drug was administered within 1 min, while a micropump was used to infuse the remaining 90% of the prescribed dose within 1 h, up to a maximum dose of <90 mg. In addition to rt-PA intravenous thrombolysis, other treatments include routine treatment of acute cerebral infarction and corresponding treatment of associated diseases (such as antihypertensive drugs for hypertension and hypoglycemic drugs for diabetes). Demographic and clinical characteristics of interest included age, sex, body mass index, medical history, smoking history, blood pressure, blood glucose levels, blood C-reactive protein (CRP) levels, blood homocysteine levels, and routine blood and biochemical indices at admission. In this study, AIS was divided into large-artery atherosclerosis (LAA), small-artery occlusion (SAO), and others (cardioembolism, unknown causes, and other causes) in accordance with the TOAST classification. In the AIS group, the degree of neurological deficits was evaluated by experienced neurologists immediately after admission using the National Institutes of Health Stroke Scale (NIHSS). The modified Rankin Scale (mRS) was used to predict patient outcomes at 3 months, with mRS scores of 0–2 and 3–6 points representing good and poor prognosis, respectively. An mRS score of 6 was defined as death. An EDTA tube was used to collect blood samples from each patient within 15 min after admission, following which rt-PA was administered for those meeting the criteria for intravenous thrombolysis. The separated plasma was stored at –80°C after centrifugation.

### Plasma levels of syndecan-1

Plasma levels of syndecan-1 were detected using a Human Syndecan-1 ELISA kit (cargo number: ab46506; batch number: GR3368280-5), in accordance with the manufacturer’s instructions, using a Bio-Tek Elx800 instrument. If a sample concentration exceeded the upper limit of the detection range, the sample was diluted and re-examined.

### Statistical analyses

SPSS (version 22.0; IBM Corp., Armonk, NY, United States) and R software (version 4.1.1) were used for statistical analyses. Continuous variables conforming to a normal distribution are reported as the mean ± SD and were compared using t-tests or analyses of variance. Variables not conforming to a normal distribution assumption are represented as the median (interquartile range) and were compared using Mann–Whitney U-tests. Correlations were analyzed using Spearman correlation coefficients. Categorical variables are reported as frequencies (%) and were compared using chi-square tests. Multivariate prognostic analysis was performed using a binary logistic regression model.

Least absolute shrinkage and selection operator (LASSO) regression was performed to screen variables relevant to the risk assessment model, including lambda.min and lambda.1se. Variables with non-zero regression coefficients were included in the final model. The LASSO logistic regression model was built using the glmpath package in R software. The risk prediction model was constructed by integrating the candidate independent risk factors. A nomogram was created based on the results of the logistic regression analysis. The consistency index (C-index), area under (AUC) the receiver operating characteristic curve (ROC), and smooth-fitting curve values were used to evaluate the discriminative ability of the nomogram. A calibration diagram was used to evaluate its consistency. The net reclassification improvement (NRI) and integrated discrimination improvement (IDI) values were used to examine the accuracy of the risk model and provide a baseline reference for model improvements. Decision curve analysis (DCA) was used to evaluate the clinical value of the line chart. *P-*values < 0.05 were considered statistically significant.

## Results

### Patient characteristics

**FIGURE 2:**
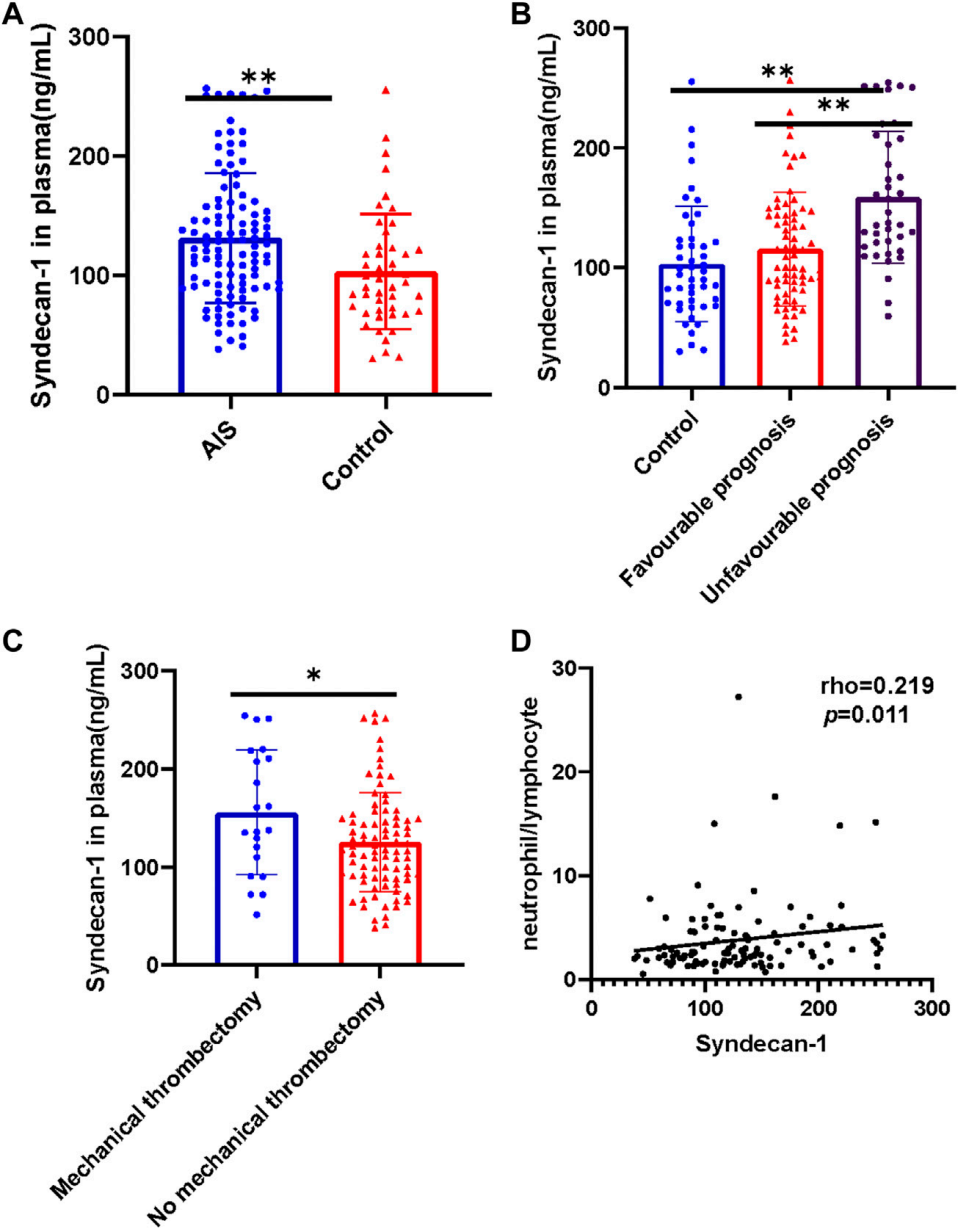
Plasma levels of syndecan-1 protein and their relationship with the neutrophil/lymphocyte ratio in different subgroups. **(A)** Comparison of plasma syndecan-1 levels between patients with AIS treated using intravenous thrombolysis and controls; **(B)** among the control group (*n* = 47), favorable prognosis group (*n* = 69), and unfavorable prognosis group (*n* = 39); **(C)** between patients treated with and without mechanical thrombectomy. **(D)** Correlation between syndecan-1 levels and neutrophil/lymphocyte ratio. The control group included healthy participants, while the AIS group included patients treated with intravenous thrombolysis (*n* = 108). *p < 0.05, **p < 0.01. AIS, acute ischemic stroke.

The distributions of age and sex were similar in the AIS (*n* = 108) and control groups (*n* = 47). Plasma levels of syndecan-1 were significantly higher in patients with AIS who had received intravenous thrombolysis than in healthy controls (t = 3.066, *p* = 0.0026, Figure 2A).

### Baseline factors influencing prognosis in patients with AIS treated with rt-PA

Based on the mRS score at 3 months, 39 (36.11%) patients had an unfavorable prognosis; among them, 29 (74.36%) were male. Older age, history of atrial fibrillation, high CRP, treatment with mechanical thrombectomy, high blood glucose, high neutrophil count, high neutrophil/lymphocyte ratio, high NIHSS score, and high blood syndecan-1 levels were more common in patients with unfavorable prognosis than in those with favorable prognosis. Patients with LAA also tended to have poor prognosis; in contrast, patients with SAO had relatively good prognosis (*p* < 0.05, Table 1).

**TABLE 1:** Baseline characteristics of AIS patients with mRS of ≤2 and that of >2 at 3 months.

	All (108)	Favourable prognosis (*n* = 69)	Unfavourable prognosis (*n* = 39)	*p* value
Demographic characteristics[n(%) OR median (IQR) OR ‾x ± s]				
Male, n (%)	85 (78.70)	56 (81.16)	29 (74.36)	0.466
Age, y, (x ± s)	64.30 ± 12.61	61.23 ± 10.92	69.72 ± 13.68	0.001
Body mass index, kg/m2	24.84 (23.85, 26.74)	25 (23.75, 26.73)	24.59 (23.85, 26.80)	0.595
Medical history [n (%)]				
Hypertension	78 (72.22)	50 (72.46)	28 (71.79)	0.556
Diabetes mellitus	37 (34.26)	23 (33.33)	14 (35.90)	0.835
Hypercholesterolemia	50 (46.30)	34 (49.28)	16 (41.03)	0.266
Coronary artery disease	21 (19.44)	13 (18.84)	8 (20.51)	0.511
Atrial fibrillation	9 (8.33)	3 (4.35)	6 (15.38)	0.046
Smoking habit	46 (42.59)	33 (47.83)	13 (33.33)	0.103
Stroke characteristics and treatment [n(%) OR median (IQR)]				
Onset-to-treatment time, h	2.20 (1.20, 3.38)	2.5 (1.20, 3.40)	2 (1.20, 3.30)	0.699
Mechanical thrombectomy	21 (19.44)	6 (8.70)	15 (38.46)	<0.001
Stroke classification[n (%)]				
LAA	65 (60.19)	36 (52.17)	29 (74.36)	0.024
SAO	36 (33.33)	28 (40.58)	8 (20.51)	0.034
Others	7 (6.48)	5 (7.25)	2 (5.13)	0.668
Complication				
sICH	2 (1.9)	1 (1.4)	2 (5.1)	0.295
General evaluation of admission[n(%) OR median (IQR) OR ‾x ± s]				
Systolic blood pressure, mm Hg	150 (140, 168)	150 (139.50, 168.00)	150 (140.00, 168.00)	0.734
Diastolic blood pressure, mm Hg	82.50 (74.25, 92.75)	82 (73.50, 93.00)	86 (75.00, 92.00)	0.885
Blood glucose concentration, g/L	7.90 (6.52, 10.30)	7.40 (6.20, 9.70)	8.90 (7.60, 11.10)	0.026
Glycated hemoglobin	6.15 (5.60, 7.30)	6 (5.60, 7.20)	6.20 (5.70, 7.30)	0.512
CRP, mg/L	2.13 (1.12, 5.89)	1.89(0.80, 4.17)	5.89 (1.75, 12.31)	<0.001
Homocysteine, μmol/L	14.80 (11.60, 17.40)	14.5 (11.40, 18.35)	14.9 (12.20, 16.10)	0.883
TC, mmol/L	1.71 (1.03, 2.70)	1.76 (1.10, 2.74)	1.7 (0.99, 2.63)	0.468
Cholesterol, mmol/L	4.62 ± 1.06	4.62 ± 1.11	4.6 ± 1.0	0.922
HDL, mmol/L	1.12 (0.94, 1.30)	1.12 (0.96, 1.36)	1.06 (0.89, 1.27)	0.359
LDL, mmol/L	2.65 ± 0.92	2.64 ± 0.97	2.7 ± 0.82	0.786
NIHSS score	5 (3, 12)	5 (3, 6.50)	12.00 (6, 18)	<0.001
WBC, ×109/L	7.38 (6.14, 9.14)	7.20 (576, 8.79)	7.87 (6.52, 10.61)	0.120
Neutrophils, ×109/L	4.71 (3.71, 6.54)	4.47 (3.53, 5.67)	5.37 (4.06, 7.95)	0.025
Lymphocytes, ×109/L	1.83 (1.32, 2.26)	1.90 (1.52, 2.33)	1.48 (1.16, 2.19)	0.053
Neutrophil-to-lymphocyte ratio	2.61 (1.84, 4.47)	2.35 (1.66, 3.46)	3.35 (2.42, 5.55)	0.010
Platelet count, ×1,000/mm3	208.5 (175, 242)	220 (177.50, 245)	201 (167, 232)	0.065
Syndecan-1,ng/mL	131.19 ± 54.35	115.60 ± 47.71	158.77 ± 54.97	<0.001

NIHSS, NIH stroke scale; IQR, interquartile range; CRP, C-reactive protein; HDL, high-density lipoprotein; LDL, low-density lipoprotein; TC, triglyceride; sICH, symptomatic intracranial hemorrhage.

### Syndecan-1 levels and prognosis

Plasma levels of syndecan-1 were significantly higher in the unfavorable prognosis group than in the favorable prognosis group (*p* < 0.001, Figure 2B and Table 1). In addition, plasma syndecan-1 levels were significantly lower in the control group than in the unfavorable prognosis group (*p* < 0.001, Figure 2B) and significantly higher in the mechanical embolectomy group than in the non-mechanical embolectomy group (t = 2.335, *p* = 0.0214, Figure 2C). Syndecan-1 levels were also positively correlated with the neutrophil-to-lymphocyte ratio (*p* = 0.011, Figure 2D).

### Variable selection

**FIGURE 3:**
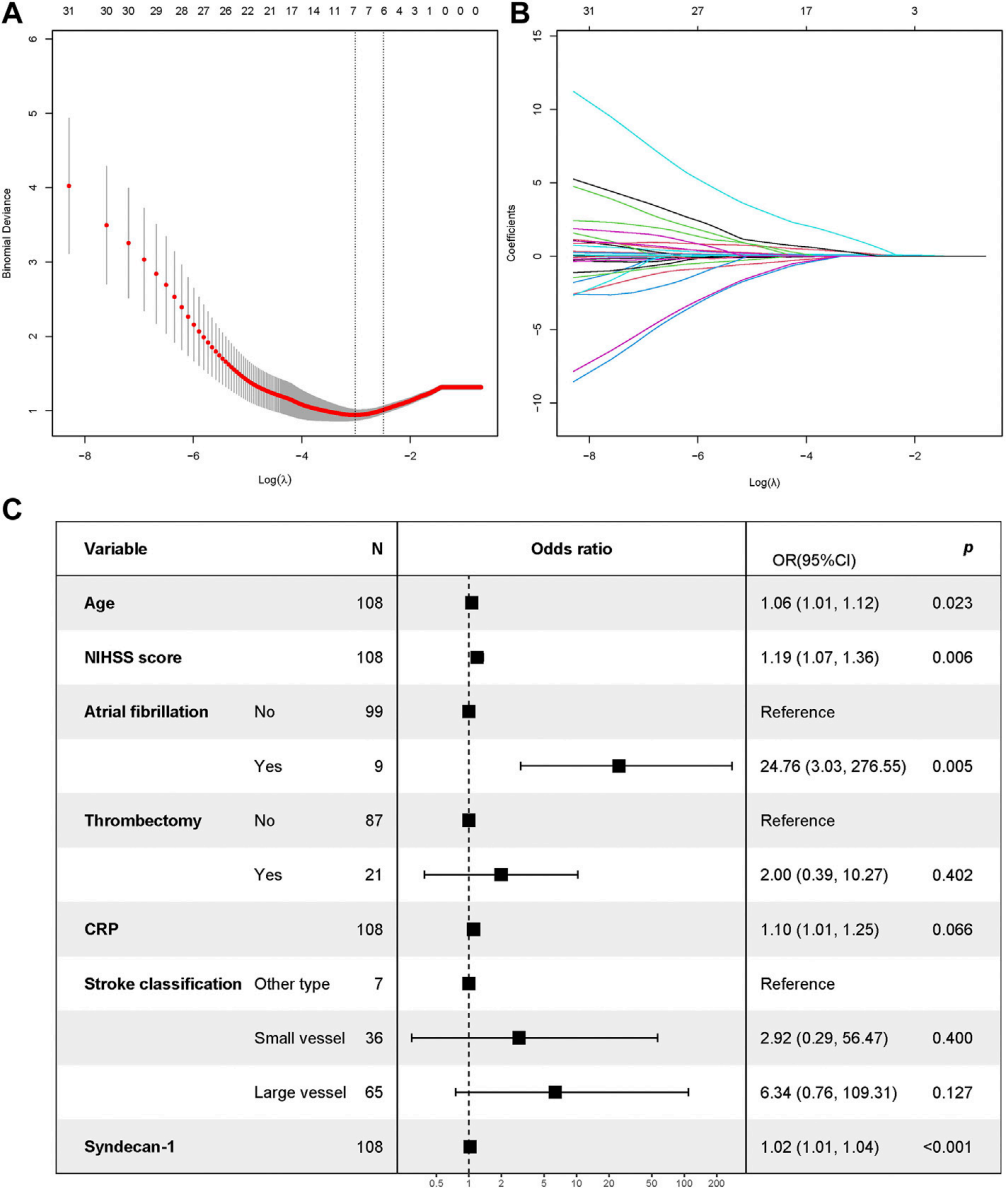
Screening of characteristic variables based on LASSO regression and forest map of factors influencing prognosis as determined using logistic regression. **(A)** The process of selecting the most suitable value for λ in the LASSO model via cross-validation method. When λ = 0.04902451 and seven parameters are selected, the LASSO regression model is most suitable; **(B)** The figure shows the characteristics of the variable coefficients. LASSO, least absolute shrinkage and selection operator; **(C)** Forest map. Note: An mRS of >2 points at 3 months was used as the dependent variable. The independent variables included age, NIHSS score, atrial fibrillation, mechanical thrombectomy, CRP, stroke classification, and syndecan-1 levels (screened out via LASSO regression). mRS, modified Rankin scale; NIHSS, National Institutes of Health Stroke Scale; CRP, C-reactive protein; LASSO, least absolute shrinkage and selection operator.

Thirty variables were screened as candidate predictors. The LASSO regression analysis yielded seven variables that most strongly correlated with adverse prognosis after thrombolysis in AIS, including age, NIHSS score, history of atrial fibrillation, mechanical thrombectomy, blood CRP level, stroke classification, and syndecan-1 level. Models with different variable configurations were tested and fitted using 10-fold cross validation. The log value of *λ* and AUC values were used for plotting. The model with the best performance and fewest independent variables corresponded to the *λ* of 0.04902451 (Figures 3A,B).

### Plasma syndecan-1 levels can better predict poor prognosis

After adjusting for age, NIHSS score, history of atrial fibrillation, mechanical thrombectomy status, blood CRP levels, stroke classification, and syndecan-1 levels, logistic regression analysis (Model 2) revealed that syndecan-1 levels remained significantly associated with unfavorable prognosis in patients with AIS treated with intravenous thrombolysis (*p* < 0.001, Table 2). After multivariate correction, the odds ratio (OR) (95% confidence interval, 95% CI) for syndecan-1 levels was 1.024 (1.010–1.038). The optimal cutoff value of plasma syndecan-1 for determining unfavorable prognosis was 102.82 ng/ml. Subsequently, syndecan-1 levels were dichotomized into ≥102.82 and <102.82 ng/ml. Syndecan-1 levels were associated with poor prognosis as a continuous variable (OR, 1.024; 95% CI, 1.010–1.038); moreover, values of ≥102.82 ng/ml were associated with poor prognosis (OR, 34.551; 95% CI, 5.408–220.732) at 3 months in patients with AIS treated with intravenous thrombolysis (Table 2).

**TABLE 2:** Syndecan-1 alone and in combination predicted the prognosis of AIS patients treated with intravenous thrombolysis.

	Model1		Model2	
	OR (95%CI)	*p* value	OR (95%CI)	*p* value
Biomarkers (as continuous variables)				
Syndecan-1^a^	1.016 (1.008, 1.025)	<0.001	1.024 (1.010, 1.038)	<0.001
Biomarkers (as categorical variables)				
Syndecan-1, ≥102.82 ng/ml^b^	11.657 (3.278, 41.461)	<0.001	34.551 (5.408, 220.732)	<0.001

MODEL 2: mRS of >2 points at 3 months was the dependent variable; covariates included age, NIHSS score, history of atrial fibrillation, mechanical thrombectomy, blood CRP, stroke classification, blood syndecan-1(screened out by the LASSO regression).

a Continuous variable.

b Categorical variable.

Based on the seven variables selected by LASSO regression, an additional binary logistic regression analysis was performed. This analysis indicated that age, NIHSS score, history of atrial fibrillation, and high plasma syndecan-1 levels were associated with poor prognosis (*p* < 0.05, Figure 3C).

### Prognostic nomogram

**FIGURE 4:**
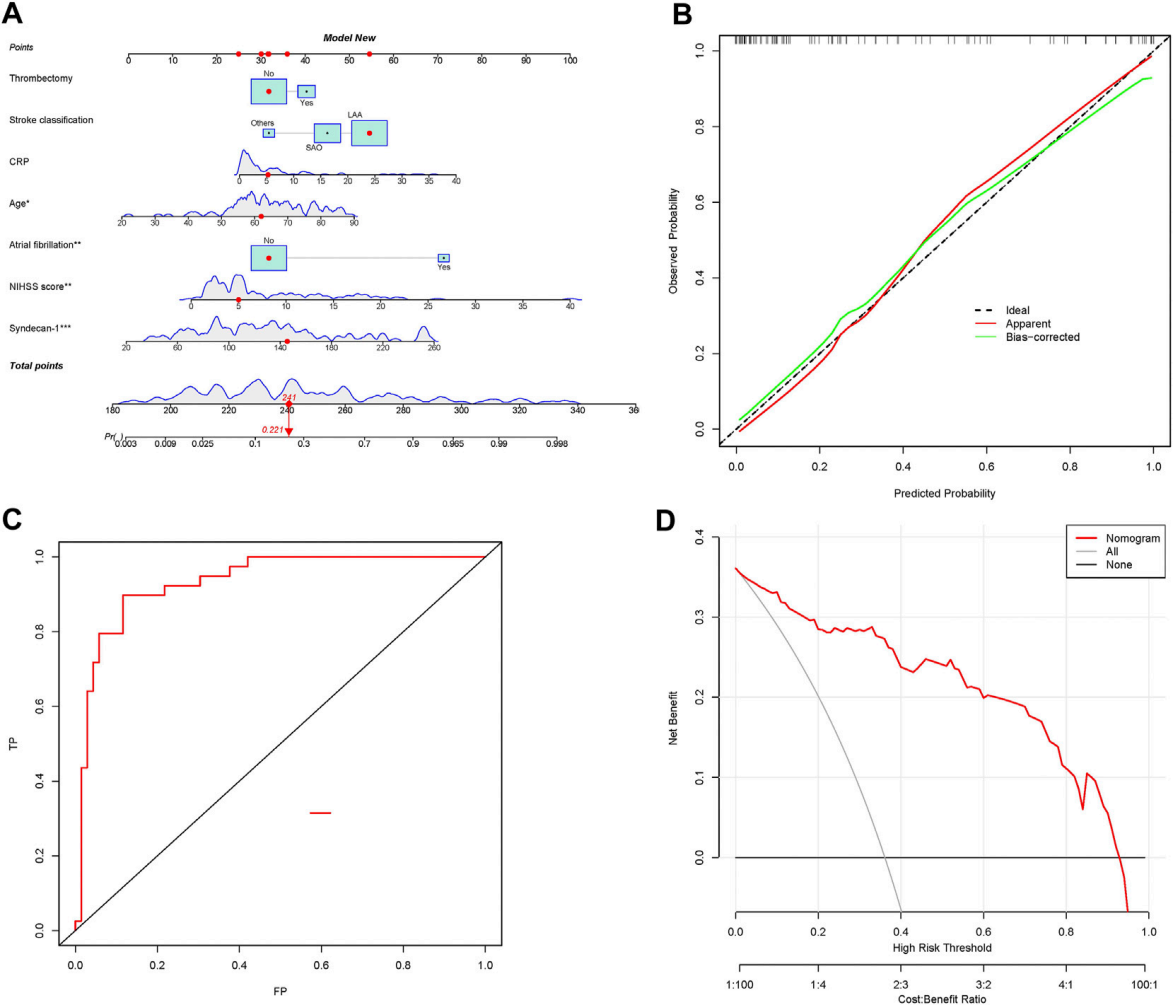
Nomograms for predicting prognosis using the risk model and evaluation of the prediction model. **(A)** Nomograms for predicting prognosis based on the risk model; **(B)** plots depicting the calibration of the risk model; **(C)** ROC plot based on nomograms for prognosis; **(D)** decision curve analysis of the model for predicting AIS prognosis following thrombolytic treatment. ROC, receiver operating characteristic.

The risk assessment models were converted into nomograms (Figure 4A). The crude and bootstrap-corrected C-index values for the nomogram were 0.935 (95% CI, 0.888–0.981) and 0.898, respectively. The predictive probability of the nomogram was in good agreement with the actual probability observed. The risk model exhibited a better fit (Figure 4B), with an AUC of 0.935 (95% CI, 0.888–0.981) (Figure 4C), as well as good discriminative ability and accuracy. The classification and continuous NRI values for the unfavorable and favorable prognosis groups were greater than 0, indicating that the predictive accuracy of the risk model was better. The IDI was 0.111 (95% CI, 0.049–0.174, *p* < 0.001), also highlighting the better predictive ability of the risk model (Table 3).

**TABLE 3:** NRI and IDI differences between the old and new models.

		Estimate	Lower	Upper	*p*
NRI (Categorical)	NRI	0.069	-0.027	0.403	0.541
	NRI+	0.026	-0.051	0.203	0.684
	NRI-	0.043	-0.028	0.252	0.554
NRI (Continuous)	NRI	0.776	0.169	1.596	0.046
	NRI+	0.385	-0.042	0.825	0.090
	NRI-	0.391	0.079	0.833	0.050
IDI		0.111	0.049	0.174	<0.001

### Risk following rt-PA treatment for AIS patients based on nomogram scores

The optimal threshold for the nomogram based on the risk model was a score of 133.92. The sensitivity, specificity, accuracy, positive predictive value (PPV), and negative predictive value (NPV) of the risk model for differentiating prognosis were 90%, 88%, 89%, 81%, and 94%, respectively (Table 4).

**TABLE 4:** Accuracy of the Prediction Score of the Nomogram for Estimating the Risk of prognosis of AIS patients treated with intravenous thrombolysis.

Variable	Risk model (OR,95% CI)
Area of ROC curve	0.93 (0.89–0.98)
Threshold	133.92
Sensitivity, %	0.90 (0.75, 0.97)
Specificity, %	0.88 (0.78, 0.95)
Accuracy	0.89 (0.81, 0.94)
PPV, %	0.81 (0.66, 0.91)
NPV, %	0.94 (0.84, 0.98)

### Clinical application

DCA quantifies the net benefit of different threshold probabilities in a dataset to determine the clinical value of a nomogram. The DCA results suggested that the risk model, which included syndecan-1 levels, exhibited a better ability to predict AIS prognosis following rt-PA treatment than the conventional model (Figure 4D). These findings suggest that the nomogram has some clinical value.

## Discussion

Ischemic stroke is the leading cause of death and disability worldwide, and its incidence is expected to increase given aging of the global population (Paul and Candelario-Jalil, 2021). Diagnostic biomarkers have been used in the context of stroke diagnosis for decades (Bsat et al., 2021), although rapid assessment and management are essential for improving patient prognosis. Syndecan-1 is a major marker of glycocalyx degradation, levels of which are elevated in the peripheral plasma of affected patients. In this study, we examined the prognostic value of syndecan-1 levels as a marker of post-thrombolysis glycocalyx injury in patients with AIS.

Previous studies have reported that ischemia/reperfusion injury can promote glycocalyx degradation (Rehm et al., 2007). However, DellaValle et al. observed no changes in syndecan-1 levels following onset in 10 patients with AIS (DellaValle et al., 2019). An animal study by Zhu et al. demonstrated that, after acute middle cerebral artery occlusion, plasma syndecan-1 levels began to rise at 1 h, peaking at 6 h, gradually decreasing thereafter, and increasing again on days 5 and 7 (Zhu et al., 2021). However, to our knowledge, no previous studies have examined the correlation between syndecan-1 levels and poor prognosis in patients with acute cerebral infarction undergoing thrombolysis. The present study is first to show that elevated plasma syndecan-1 levels may be associated with poor prognosis in patients with AIS treated with intravenous thrombolysis. Moreover, adding syndecan-1 levels to the conventional risk assessment model increased model discrimination and accuracy. These findings indicate that syndecan-1 levels may play an important role in the pathophysiology of AIS, highlighting their potential as a target in the diagnosis and treatment of patients with AIS following intravenous thrombolysis.

Previous studies have demonstrated that inflammation plays a role in the development of AIS (Gasbarrino et al., 2019; Rivera-Caravaca et al., 2019) and may affect patient prognosis. High levels of circulating syndecan-1 may contribute to such inflammation (Johansson et al., 2011). Stroke severity is typically evaluated using the NIHSS, with higher scores generally indicating a worse prognosis following AIS. In addition, age has been identified as a risk factor for poor AIS prognosis following treatment with intravenous thrombolysis. Age also increases the risk of ischemic stroke and reduces the likelihood of recovery following damage to the neurovascular unit (Soriano-Tárraga et al., 2017). In the present study, patients with AIS that underwent concurrent mechanical thrombectomy exhibited poorer prognosis than their counterparts, which may have been related to the severity of cerebrovascular disease and the extent of the infarction. Atrial fibrillation has also been associated with poor prognosis and may be a marker of poor cardiac function. Patients with macrovascular disease may present with cerebral infarction of a larger extent, resulting in more severe neurological deficits and poor prognosis.

In the univariate analysis, plasma syndecan-1 levels were higher among patients with poor prognosis after intravenous thrombolysis than among those with good prognosis. Multivariate logistic regression analysis verified that syndecan-1 levels were associated with poor prognosis. Furthermore, addition of syndecan-1 levels to the conventional risk prediction model significantly improved its prognostic efficiency. In this study, a nomogram was used to visualize the prediction model; the AUC and fitting curve were used to determine the discrimination and consistency of the prediction model. The NRI and IDI values were used to evaluate model accuracy. Overall, the new risk model incorporating syndecan-1 exhibited better discriminative ability, consistency, and accuracy than the conventional model. DCA revealed that the new model had greater clinical value than the conventional model. This study is first to report that syndecan-1 levels in patients with AIS undergoing intravenous thrombolysis may predict prognosis, highlighting its potential value as a biomarker for risk stratification. Improved stratification may aid in identifying patients at the greatest risk for poor prognosis, which may in turn facilitate early diagnosis and treatment, thereby improving outcomes.

Presently, rt-PA thrombolysis is the only AIS treatment approved by the Food and Drug Administration. However, the clinical use of rt-PA is limited to less than 5% of patients because of the narrow treatment window (Kleindorfer et al., 2008). Delayed rt-PA treatment increases the risk of cerebral edema and hemorrhage, increasing the risk of mortality in this patient group (Mohr, 2000). In general, rt-PA may trigger bleeding by weakening the blood–brain barrier. Upon entering the brain, rt-PA induces cytokine production and activates MMP-9 (Tsuji et al., 2005). Research has indicated that use of hyperbaric oxygen with rt-PA can reduce the extent of rt-PA-related bleeding, likely by strengthening the blood–brain barrier (Kim et al., 2005). Preventing damage to the blood–brain barrier can help protect the brain and reduce the risk of tPA-related adverse events, thereby extending the treatment window. In rodent experiments, rt-PA has been shown to aggravate ischemia-induced damage to the blood–brain barrier by enhancing the proteolytic activity of MMP-9 (Pfefferkorn and Rosenberg, 2003; Wang et al., 2003). Studies involving human patients with stroke have demonstrated that high plasma MMP-9 levels before treatment are more likely to result in cerebral hemorrhage complications after rt-PA therapy (Zhong et al., 2019). MMP inhibitors may prevent the premature opening of the blood–brain barrier and reduce the risk of bleeding and death when rt-PA is administered during stroke (Lapchak et al., 2000). Since glycocalyx is an important component of the blood–brain barrier, inhibiting glycocalyx degradation may protect the blood–brain barrier and reduce rates of bleeding, especially in patients with nerve damage caused by rt-PA. This may help to reduce mortality following rt-PA thrombolysis and safely extend the treatment window. Glycocalyx degradation inhibitors combined with rt-PA thrombolytic therapy may therefore help to improve the treatment of acute cerebral infarction.

## Conclusion

The current findings demonstrate that incorporating plasma syndecan-1 levels may help to improve the risk stratification of patients with acute cerebral infarction undergoing intravenous thrombolysis. Plasma syndecan-1 levels combined with clinical and imaging test results may therefore aid in identifying patients at the greatest risk for poor outcomes. However, large-scale, multi-center studies are required to validate the present findings.

## Data Availability

The raw data supporting the conclusion of this article will be made available by the authors, without undue reservation.

## References

[B1] Bsat S. Halaoui A. Kobeissy F. Moussalem C. El Houshiemy M. N. Kawtharani S. (2021). Acute ischemic stroke biomarkers: a new era with diagnostic promise. Acute Med. Surg. 8 (1), e696. 10.1002/ams2.696 34745637 PMC8552525

[B2] Chronopoulos A. Thorpe S. D. Cortes E. Lachowski D. Rice A. J. Mykuliak V. V. (2020). Syndecan-4 tunes cell mechanics by activating the kindlin-integrin-RhoA pathway. Nat. Mat. 19 (6), 669–678. 10.1038/s41563-019-0567-1 PMC7260055 31907416

[B3] DellaValle B. Hasseldam H. Johansen F. F. Iversen H. K. Rungby J. Hempel C. (2019). Multiple soluble components of the glycocalyx are increased in patient plasma after ischemic stroke. Stroke 50 (10), 2948–2951. 10.1161/STROKEAHA.119.025953 31409270

[B4] Faria-Ramos I. Poças J. Marques C. Santos-Antunes J. Macedo G. Reis C. A. (2021). Heparan sulfate glycosaminoglycans: (Un)Expected allies in cancer clinical management. Biomolecules 11 (2), 136. 10.3390/biom11020136 33494442 PMC7911160

[B5] Gasbarrino K. Hafiane A. Zheng H. Daskalopoulou S. S. (2019). Intensive statin therapy compromises the adiponectin-AdipoR pathway in the human monocyte-macrophage lineage. Stroke 50 (12), 3609–3617. 10.1161/STROKEAHA.119.026280 31648632

[B6] Johansson P. I. Stensballe J. Rasmussen L. S. Ostrowski S. R. (2011). A high admission syndecan-1 level, a marker of endothelial glycocalyx degradation, is associated with inflammation, protein C depletion, fibrinolysis, and increased mortality in trauma patients. Ann. Surg. 254 (2), 194–200. 10.1097/SLA.0b013e318226113d 21772125

[B7] Kim H. B. Soh S. Kwak Y. L. Bae J. C. Kang S. H. Song J. W. (2020). High preoperative serum syndecan-1, a marker of endothelial glycocalyx degradation, and severe acute kidney injury after valvular heart surgery. J. Clin. Med. 9 (6), E1803. 10.3390/jcm9061803 32531891 PMC7356050

[B8] Kim H. Y. Singhal A. B. Lo E. H. (2005). Normobaric hyperoxia extends the reperfusion window in focal cerebral ischemia. Ann. Neurol. 57 (4), 571–575. 10.1002/ana.20430 15786465

[B9] Kleindorfer D. Lindsell C. J. Brass L. Koroshetz W. Broderick J. P. (2008). National US estimates of recombinant tissue plasminogen activator use: ICD-9 codes substantially underestimate. Stroke 39 (3), 924–928. 10.1161/STROKEAHA.107.490375 18239184

[B10] Lapchak P. A. Chapman D. F. Zivin J. A. (2000). Metalloproteinase inhibition reduces thrombolytic (tissue plasminogen activator)-induced hemorrhage after thromboembolic stroke. Stroke 31 (12), 3034–3040. 10.1161/01.str.31.12.3034 11108768

[B11] Lu R. Sui J. Zheng X. L. (2020). Elevated plasma levels of syndecan-1 and soluble thrombomodulin predict adverse outcomes in thrombotic thrombocytopenic purpura. Blood Adv. 4 (21), 5378–5388. 10.1182/bloodadvances.2020003065 33141886 PMC7656933

[B12] Mohr J. P. (2000). Thrombolytic therapy for ischemic stroke: from clinical trials to clinical practice. JAMA 283 (9), 1189–1191. 10.1001/jama.283.9.1189 10703782

[B13] National Institute of Neurological Disorders and Stroke rt-PA Stroke Study Group (1995). Tissue plasminogen activator for acute ischemic stroke. N. Engl. J. Med. 333 (24), 1581–1587. 10.1056/NEJM199512143332401 7477192

[B14] Paul S. Candelario-Jalil E. (2021). Emerging neuroprotective strategies for the treatment of ischemic stroke: an overview of clinical and preclinical studies. Exp. Neurol. 335, 113518. 10.1016/j.expneurol.2020.113518 33144066 PMC7869696

[B15] Pfefferkorn T. Rosenberg G. A. (2003). Closure of the blood-brain barrier by matrix metalloproteinase inhibition reduces rtPA-mediated mortality in cerebral ischemia with delayed reperfusion. Stroke 34 (8), 2025–2030. 10.1161/01.STR.0000083051.93319.28 12855824

[B16] Rehm M. Bruegger D. Christ F. Conzen P. Thiel M. Jacob M. (2007). Shedding of the endothelial glycocalyx in patients undergoing major vascular surgery with global and regional ischemia. Circulation 116 (17), 1896–1906. 10.1161/CIRCULATIONAHA.106.684852 17923576

[B17] Rivera-Caravaca J. M. Marín F. Vilchez J. A. Galvez J. Esteve-Pastor M. A. Vicente V. (2019). Refining stroke and bleeding prediction in atrial fibrillation by adding consecutive biomarkers to clinical risk scores. Stroke 50 (6), 1372–1379. 10.1161/STROKEAHA.118.024305 31084333

[B18] Soriano-Tárraga C. Mola-Caminal M. Giralt-Steinhauer E. Ois A. Rodriguez-Campello A. Cuadrado-Godia E. (2017). Biological age is better than chronological as predictor of 3-month outcome in ischemic stroke. Neurology 89 (8), 830–836. 10.1212/WNL.0000000000004261 28733340

[B19] Stanimirovic D. B. Friedman A. (2012). Pathophysiology of the neurovascular unit: disease cause or consequence. J. Cereb. Blood Flow. Metab. 32 (7), 1207–1221. 10.1038/jcbfm.2012.25 22395208 PMC3390807

[B20] Tsuji K. Aoki T. Tejima E. Arai K. Lee S. R. Atochin D. N. (2005). Tissue plasminogen activator promotes matrix metalloproteinase-9 upregulation after focal cerebral ischemia. Stroke 36 (9), 1954–1959. 10.1161/01.STR.0000177517.01203.eb 16051896

[B21] van Vliet E. A. Aronica E. Gorter J. A. (2014). Role of blood-brain barrier in temporal lobe epilepsy and pharmacoresistance. Neuroscience 277, 455–473. 10.1016/j.neuroscience.2014.07.030 25080160

[B22] Wang L. Huang X. Kong G. Xu H. Li J. Hao D. (2016). Ulinastatin attenuates pulmonary endothelial glycocalyx damage and inhibits endothelial heparanase activity in LPS-induced ARDS. Biochem. Biophys. Res. Commun. 478 (2), 669–675. 10.1016/j.bbrc.2016.08.005 27498004

[B23] Wang X. Lee S. R. Arai K. Lee S. R. Tsuji K. Rebeck G. W. (2003). Lipoprotein receptor-mediated induction of matrix metalloproteinase by tissue plasminogen activator. Nat. Med. 9 (10), 1313–1317. 10.1038/nm926 12960961

[B24] Yao W. Rose J. L. Wang W. Seth S. Jiang H. Taguchi A. (2019). Syndecan 1 is a critical mediator of macropinocytosis in pancreatic cancer. Nature 568 (7752), 410–414. 10.1038/s41586-019-1062-1 30918400 PMC6661074

[B25] Yepes M. Sandkvist M. Moore E. G. Bugge T. H. Strickland D. K. Lawrence D. A. (2003). Tissue-type plasminogen activator induces opening of the blood-brain barrier via the LDL receptor-related protein. J. Clin. Invest. 112 (10), 1533–1540. 10.1172/JCI19212 14617754 PMC259131

[B26] Zhong Di Zhang Shuting Wu Bo (2019). Interpretation of ' guidelines for the diagnosis and treatment of acute ischemic stroke in china 2018. Chin. J. Mod. Neurology 19 (11), 897–901.

[B27] Zhu J. Li Z. Ji Z. Wu Y. He Y. Liu K. (2021). Glycocalyx is critical for blood-brain barrier integrity by suppressing caveolin1-dependent endothelial transcytosis following ischemic stroke. Brain Pathol. 32, e13006. 10.1111/bpa.13006 34286899 PMC8713524

[B28] Zhu J. Wan Y. Xu H. Wu Y. Hu B. Jin H. (2019). The role of endogenous tissue-type plasminogen activator in neuronal survival after ischemic stroke: friend or foe. Cell. Mol. Life Sci. 76 (8), 1489–1506. 10.1007/s00018-019-03005-8 30656378 PMC11105644

